# The Untimely Popping Phial: Poppers as an Unusual Cause of Skin Corrosion of the Thigh

**DOI:** 10.1155/2020/2058624

**Published:** 2020-01-30

**Authors:** Fabienne Moret, Gregor Lindner, Bertram K. Woitok

**Affiliations:** Department of General Internal and Emergency Medicine, Buergerspital Solothurn, Solothurn, Switzerland

## Abstract

**Background:**

Alkyl nitrites or “poppers” are widely used as sex-drugs due to their aphrodisiac and muscle relaxant effects. We describe the rare case of a large-sized dermatitis after direct skin contact with the poppers-fluid in a poppers user. *Case Presentation*. A 52-year-old patient presented to the emergency department due to burning pain on his proximal right thigh and scrotum. Clinical examination showed an 8 × 5 cm measuring burning wound resembling lesion. During further history the patient mentioned that the day before presentation a “poppers” phial unintentionally opened in his pocket and the fluid leaked.

**Conclusions:**

The present case shows severe skin defects after skin-contact with alkyl nitrates in a “poppers” user. Maculopathy and methemoglobinemia are prominent unwanted side effects of “poppers” use. However, our report demonstrates that attention should also be paid to potential harm for the skin.

## 1. Case

### 1.1. Background

Alkyl nitrites, or “poppers” in lay language, are easily available and widely used volatile nitrites, which are consumed via inhalation for their aphrodisiac and muscle-relaxant effects [1]. Originally, the substances were used for antianginal therapy in patients with coronary artery disease due to their vasodilating effects [2,3]. Besides the plausible hypothesis that “poppers” use is associated with high-risk sexual behavior [4], the most prominent unwanted side effects of its use are methemoglobinemia [5] and maculopathy [6]. Methemoglobinemia is usually caused by intentional or unintentional oral ingestion of the substance and treated by administration of methylene blue. Maculopathy is characterized by direct damage to the photoreceptors after inhalation of the drug that was described to be potentially reversible after sustaining drug use [6]. Skin-related unwanted side effects of “poppers” used have been described in literature; however, they are described mainly as facial allergic dermatitis caused by inhalation of the drug.

We present the case of severe skin lesions due to direct skin contact with “poppers” fluid.

### 1.2. Case Presentation

**Figure 1:**
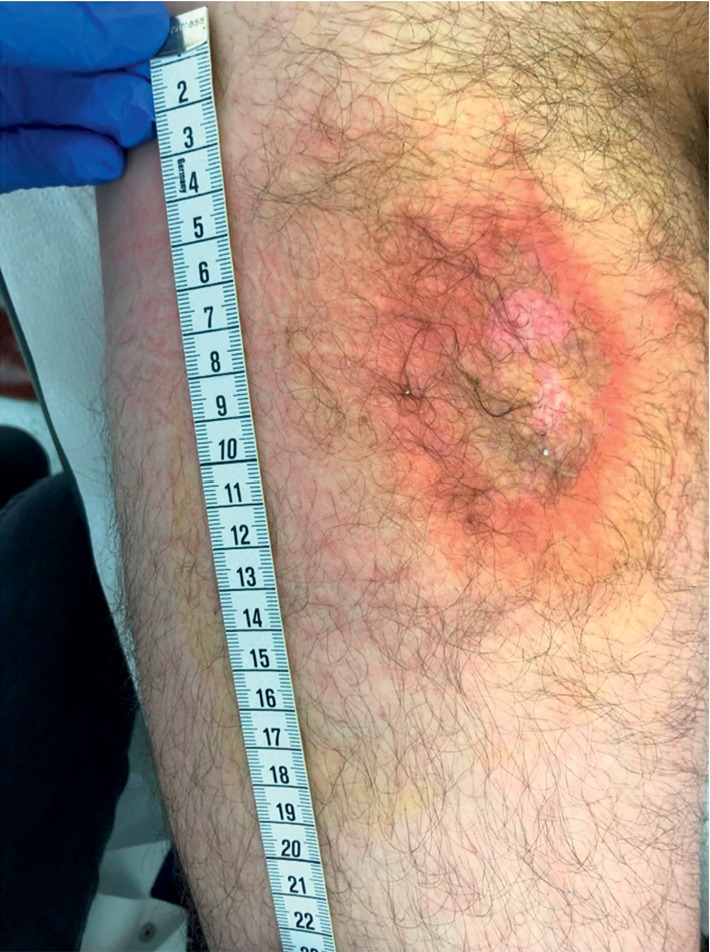
Skin lesion of the patient on the right proximal thigh.

A 52-year-old male patient presented to our emergency department due to burning pain on his right proximal thigh starting the day before. The patient reported he noticed a wet wound on the anterior right thigh. Clinical examination showed a healthy appearing man in no distress with regular vital signs and normal body temperature. Inspection of the skin revealed an 8 × 5 cm measuring wound on the anterior proximal thigh of the right leg with reddish wound borders (Figure 1). Moreover, a small wound was found on the adjacent part of the patients' scrotum, which the patient then reported to be painful.

Laboratory investigations revealed a normal red and white blood count as well as normal serum creatinine and C-reactive protein levels.

Concerning the etiology of the skin lesion, the patient reported having received a phial of “poppers” from a friend of him. During a walk, he carried the phial in the right pocket of his pants, and apparently the locker opened and the fluid leaked. The concerning pocket of the pants clearly showed a brownish discoloration. The lesions on the thigh and scrotum were thus considered to be induced by the poppers-fluid.

Since the wound appeared clean and only minimally inflamed, we decided to thoroughly disinfect the wound and applied a dry gaze. The patient was instructed to keep the wound dry and clean. On outpatient follow-up, two days after the initial consultation, the wound showed regular healing without signs of infection and improving pain. Further follow-up was planned with the primary-care physician.

## 2. Discussion and Conclusions

The present case is to our knowledge the first in literature describing clinically relevant skin corrosion after direct contact with “poppers” fluid.

Alkyl nitrites are volatile nitrites, which were originally used to treat angina pectoris, but were discarded in favour of longer acting agents [2]. They were distributed in glass vials, which had to be broken to inhale the therapeutic agent, and hence the slang term “poppers” appeared. Today, they are mainly used by men who have sex with men to relax the anal sphincter and to enhance the intensity of sexual intercourse. Alkyl nitrites are usually sold labelled as room odors or cleaning agents with consequent abuse by customers.

Usual side effects are reversible macula degeneration, but hemolytic anemia has also been reported [1,6]. Upon oral ingestion they potentially lead to methhemoglobinemia which needs to be treated with methylene blue [5]. Contact dermatitis secondary to alkyl nitrates has been described in the literature, but in clinical practice, the causation is rarely as obvious as in the case presented. Suspicion should be raised in unclear skin lesions in men who have sex with men and people committed to a hedonistic lifestyle [7].

In conclusion, this is the first case to our knowledge describing clinically relevant skin corrosion after direct contact with alkyl nitrates.
